# Development of Dosimetric Verification System for Patient-Specific Quality Assurance of High-Dose-Rate Brachytherapy

**DOI:** 10.3389/fonc.2021.647222

**Published:** 2021-03-09

**Authors:** Sang-Won Kang, Jin-Beom Chung, Kyeong-Hyeon Kim, Chang Heon Choi, Seonghee Kang, Dong-Seok Shin, Woong Cho, Kuen-Yong Eom, Hae-Jin Park, Jin-Young Kim, Changhoon Song, In Ah Kim, Jae-Sung Kim, Tae Suk Suh, Justin C. Park

**Affiliations:** ^1^Department of Biomedical Engineering, Department of Biomedicine and Health Sciences, College of Medicine, The Catholic University of Korea, Seoul, South Korea; ^2^College of Medicine, Research Institute of Biomedical Engineering, The Catholic University of Korea, Seoul, South Korea; ^3^Department of Radiation Oncology, Seoul National University Bundang Hospital, Seongnam, South Korea; ^4^Department of Radiation Oncology, Seoul National University Hospital, Seoul, South Korea; ^5^Department of Radiation Oncology, Seoul National University Boramae Medical Center, Seoul, South Korea; ^6^Department of Radiation Oncology, Ajou University School of Medicine, Suwon, South Korea; ^7^Departments of Radiation Oncology, Dongnam Institute of Radiological & Medical Sciences, Busan, South Korea; ^8^Department of Radiation Oncology, University of Texas Southwestern Medical Center, Dallas, TX, United States

**Keywords:** patient-specific quality assurance, dosimetric verification, high-dose-rate brachytherapy, film measurement, independent dose calculation

## Abstract

**Purpose:** The aim of this study was to develop a dosimetric verification system (DVS) using a solid phantom for patient-specific quality assurance (QA) of high-dose-rate brachytherapy (HDR-BT).

**Methods:** The proposed DVS consists of three parts: dose measurement, dose calculation, and analysis. All the dose measurements were performed using EBT3 film and a solid phantom. The solid phantom made of acrylonitrile butadiene styrene (ABS, density = 1.04 g/cm^3^) was used to measure the dose distribution. To improve the accuracy of dose calculation by using the solid phantom, a conversion factor [CF(r)] according to the radial distance between the water and the solid phantom material was determined by Monte Carlo simulations. In addition, an independent dose calculation program (IDCP) was developed by applying the obtained CF(r). To validate the DVS, dosimetric verification was performed using gamma analysis with 3% dose difference and 3 mm distance-to-agreement criterion for three simulated cases: single dwell position, elliptical dose distribution, and concave elliptical dose distribution. In addition, the possibility of applying the DVS in the high-dose range (up to 15 Gy) was evaluated.

**Results:** The CF(r) between the ABS and water phantom was 0.88 at 0.5 cm. The factor gradually increased with increasing radial distance and converged to 1.08 at 6.0 cm. The point doses 1 cm below the source were 400 cGy in the treatment planning system (TPS), 373.73 cGy in IDCP, and 370.48 cGy in film measurement. The gamma passing rates of dose distributions obtained from TPS and IDCP compared with the dose distribution measured by the film for the simulated cases were 99.41 and 100% for the single dwell position, 96.80 and 100% for the elliptical dose distribution, 88.91 and 99.70% for the concave elliptical dose distribution, respectively. For the high-dose range, the gamma passing rates in the dose distributions between the DVS and measurements were above 98% and higher than those between TPS and measurements.

**Conclusion:** The proposed DVS is applicable for dosimetric verification of HDR-BT, as confirmed through simulated cases for various doses.

## Introduction

High dose-rate brachytherapy (HDR-BT) can be used to effectively treat cancer by delivering high doses of radiation locally and improving both target coverage and organ sparing. Its effectiveness is remarkably high for large clinical targets with complex topologies (1). However, the risks during HDR-BT are higher than those during external beam radiotherapy when a treatment accident occurs and the causing error is not immediately identified. Errors in HDR-BT have been reported in the previous studies (2–5), and they are mainly caused by inappropriate radiation source selection, source strength units, entry into a treatment planning system (TPS), and source dwell position.

To prevent such errors, recommendations and guidelines for quality assurance (QA) of HDR-BT have been proposed (6–10). Most existing QA procedures are performed as basic tests of specific dosimetric parameters and include measuring the source activity, verifying the source position, and checking the timer accuracy and linearity using well-type chambers, special rulers, and established techniques for teletherapy sources. Although existing QA procedures are suitable for conventional HDR-BT, they may fail for the latest HDR-BT interventions. This is because the accuracy assessment paradigm for HDR-BT has shifted from determining the conventional source dwell pattern using 2D imaging to patient-specific 3D image-based optimization and inverse planning. Therefore, QA of HDR-BT based on dosimetric verification has been addressed recently (11–14).

Qi et al. (11) performed treatment plan verification for HDR-BT using a specific water phantom and metal–oxide–semiconductor field-effect transistor. Palmer et al. (12) proposed dose distribution verification using radiochromic film dosimetry in clinical brachytherapy and found that the EBT3 GAFCHROMIC^TM^ film can perform accurate dose verification in HDR-BT owing to its excellent spatial resolution, tissue equivalence, and self-development properties. In addition, they compared planned with measured dose distributions using an in-house water phantom. However, these methods can only be used to measure dose in a specific water phantom. To overcome this limitation, various methods have been proposed to replace water phantoms with solid phantoms by adopting water equivalent materials (13, 14). Meigooni et al. (13) obtained the conversion factor (CF) for media between water and various materials, such as solid water, polystyrene, and acrylic, using Monte Carlo (MC) simulations. Aldelaijan et al. (14) used the CF to perform dosimetric QA of HDR-BT with the Solid Water^TM^ phantom replacing a water phantom. However, the CF has not been applied to compare the planned and measured dose distributions but only to evaluate point doses due to limitations of commercial TPSs. In fact, dosimetric verification of HDR-BT using a commercially available TPS generally provides dose distributions by using embedded algorithms based on the AAPM (American Association of Physicists in Medicine) Task Group 43U1 report (15). These algorithms assume that the overall dose calculation is done in water, and access to the algorithm is restricted. Therefore, several previous studies have been either limited to specific water phantoms to measure and verify the dose distribution in the treatment plan or focused on evaluation of point doses by applying the CF (11–14).

In this study, a dosimetric verification system (DVS) intended for the solid phantom was developed for HDR-BT. First, we fabricated a solid phantom that can be used for film dosimetry and calculate CF(*r*), a CF value that is a function of the radial distance between water and the phantom material. Second, we developed an independent dose calculation program (IDCP) to apply the obtained CF(*r*). Third, we compared the gamma evaluation for the dose distribution calculated by the IDCP with the measured dose distribution obtained by using EBT3 film in the solid phantom. Thus, this study aimed to demonstrate the feasibility of the proposed DVS as a patient-specific QA tool for HDR-BT through various simulated cases.

## Materials and Methods

### Simple Solid Phantom

**Figure 1:**
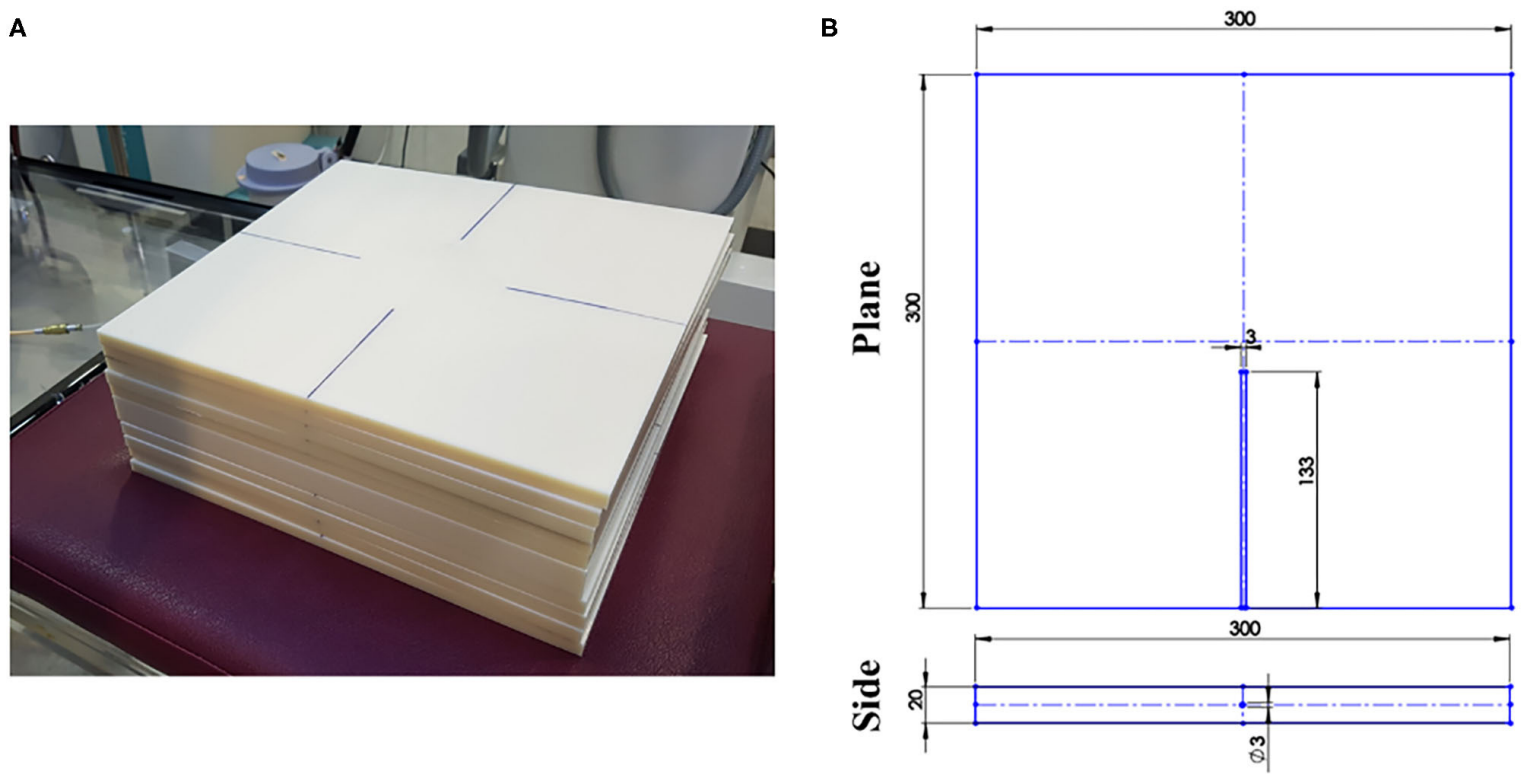
**(A)** Manufactured solid phantom and **(B)** diagram of catheter-inserted slab in phantom.

Figure 1 shows the solid phantom made of acrylonitrile butadiene styrene (ABS, density: 1.04 g/cm^3^) used to measure the dose distribution. The solid phantom comprises a normal slab (dimensions 30 × 30 × 1 cm^3^) and a catheter-inserted slab (dimensions 30 × 30 × 2 cm^3^). The catheter-inserted slab contains a parallel hole in the center of one side and the depth of the hole is 133 mm. The hole accommodated the catheter of 3 mm, and the end of the hole was attached to the catheter tip.

### Conversion Factor Between Water and ABS

The CF(*r*) between water and ABS was determined as a function of radial distance *r* by MC simulations using the Geant4 Application for Tomographic Emission (GATE4, Version 8.1). For the MC simulations, the ^192^Ir mHDR-v2 source was modeled as described by Granero et al. (16), and the calculated grid size was 1 × 1 × 1 mm^3^. The electromagnetic standard model, option3 (Emstandard_opt3), was selected from the physics engine list. The virtual phantoms with water and ABS had the same dimensions, 30 × 30 × 20 cm^3^. The ABS was composed of 45.5% carbon, 51.5% hydrogen, and 3% nitrogen (17). The CF(*r*) was obtained by the ratio of the dose profiles for ABS and water, ABS/water, along the vertical axis with respect to the source.

### Independent Dose Calculation Program (IDCP)

The proposed IDCP combines the obtained CF(*r*) to calculate the dose in the solid phantom. The IDCP is based on the AAPM Task Group 43U1 report and the dose calculation algorithm proposed in our previous study (18). The line source model is implemented in the IDCP, and the equation of the model is expressed as

(1) $$D\left(r,\;\theta \right)\;=\;S_{K}\;\cdot \;\Lambda \;\cdot \;\frac{G_{L}\left(r,\;\theta \right)}{G_{L}\left(r_{0},\;\theta_{0}\right)}\;\cdot \;g_{L}\left(r\right)\;\cdot \;F\left(r,\;\theta \right)\;\cdot \;CF\left(r\right),$$

where *r* is the distance from the center of the source, θ is the polar angle between the source longitudinal axes, *r*_0_ and θ_0_ are the reference distance (1 cm) and angle (90°), respectively, and the air-kerma strength (unit U: cGy·cm^2^·h^−1^), dose-rate constant (unit: cGy·h^−1^·U^−1^), geometry factor, radial dose function, and anisotropy function are denoted as *S*_K_, Λ, G(*r*, θ), g(*r*), and F(*r*, θ), respectively. The values of *S*_K_ and Λ are obtained from the TPS, and those of g(*r*) and F(*r*, θ) are provided by the source manufacturer. Then, CF(*r*) can be obtained through the MC simulations.

### Validation of the Developed Dosimetric Verification System Using Film Dosimetry

**Figure 2:**
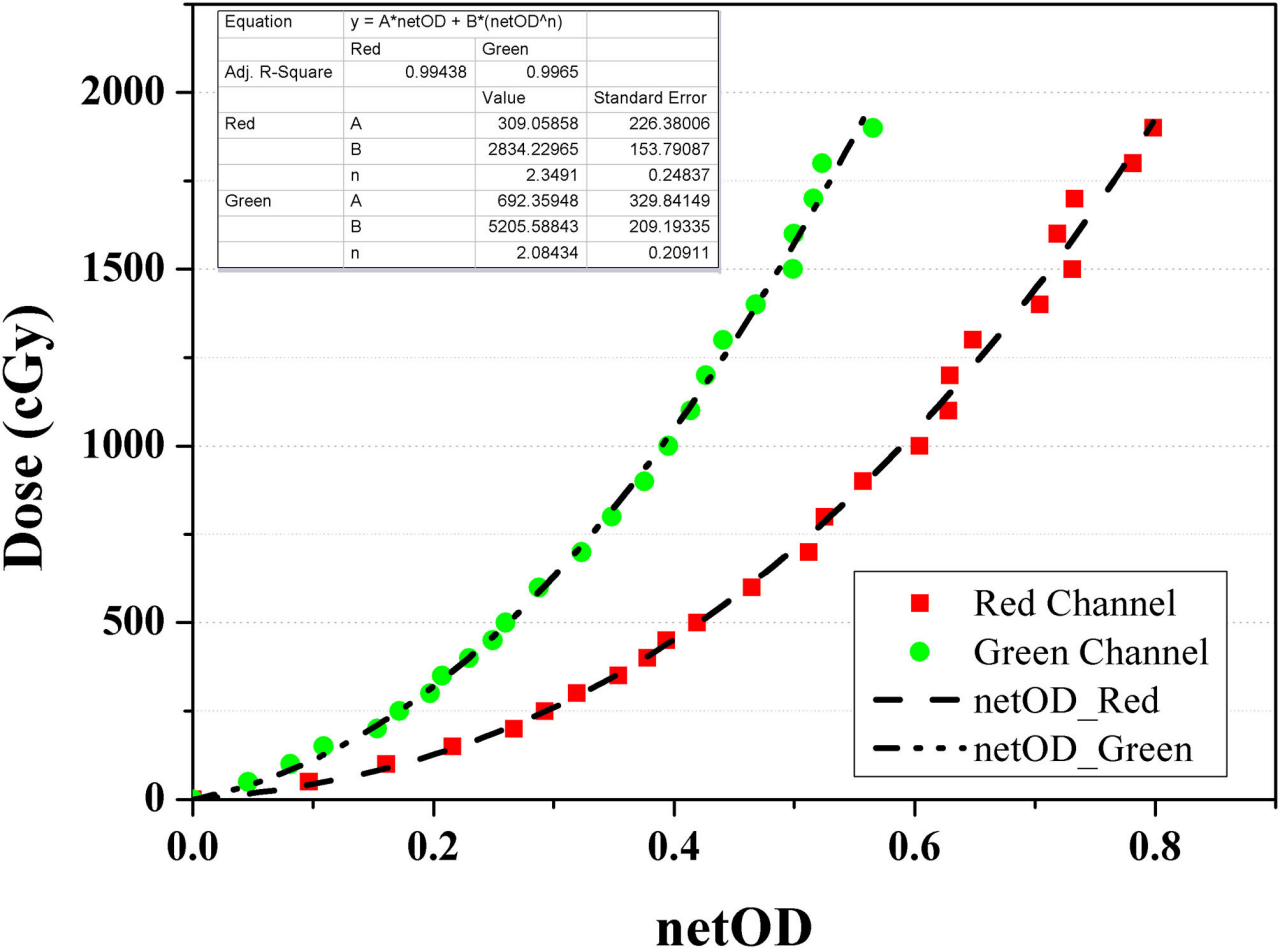
Net optical density curve for GAFCHROMIC EBT3 film calibration using red (square) and green (circle) channel.

All the measurements were performed using EBT3 film (Ashland ISP Advanced Materials, NJ, USA) from a single batch. Before film dosimetry, doses of 0–19 Gy were irradiated using a 6 MV external photon beam generated by VitalBeam (Varian Medical Systems, Palo Alto, CA, USA) to calibrate the film. The net optical density (netOD) curve was obtained from two channels (i.e., red and green), as shown in Figure 2, because each channel has a different sensitivity depending on the dose range. Specifically, the red channel provides the optimal performance at doses below 10 Gy, whereas the green channel is adaptable to high doses above 10 Gy. The netOD curve was applied with an appropriate selection for the dose range in the simulated cases.

The measurements for DVS validation were performed 1 cm below the source. The radiation exposure was implemented by NUCLETRON microSelectron Afterloader (Elekta, Stockholm, Sweden) with an ^192^Ir mHDR-v2 source. The measurements adhered to the procedure for handling EBT3 film recommended by the AAPM Task Group 53 report. The DVS validation was executed by dosimetric verifications for three cases: First, the single dwell position was measured to evaluate the feasibility of the CF(*r*) and DVS operation. The source was in the catheter tip in the phantom, and doses of 4 Gy were irradiated 1 cm below the source. The corresponding dose distributions and profiles according to the angle were then measured. Second, the elliptical dose distributions at the same dwell-time at a linear dwell position of 5 mm was measured. Third, the concave elliptical dose distribution was established using different dwell-times, and the corresponding dose distribution was measured. We also evaluated the applicability of the DVS for high-dose by performing dosimetric verification for various high-dose ranges including 9.5, 10.75, 13.5, and 15 Gy.

Each dose distribution on the same measurement plane was calculated using the Oncentra Brachy software (Elekta) and the proposed IDCP. The gamma analysis developed by Low et al. (19) was used to evaluate the measured and calculated dose distributions using global normalization with a 3% dose difference and 3 mm distance to agreement (3%/3 mm criterion).

## Results

### Percentage Dose and CF(r) for Water and ABS Phantom Using MC Simulation

**Figure 3:**
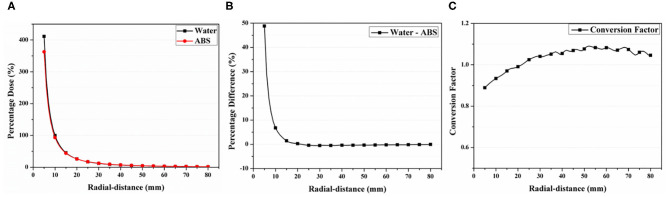
**(A)** Percentage dose profiles calculated by Monte Carlo simulation for water and acrylonitrile butadiene styrene (ABS) material. **(B)** Percentage difference between calculated dose profiles and **(C)** conversion factor obtained using ABS/water ratio.

Figure 3 shows the calculated percentage dose profiles according to the radial distances obtained from the virtual water and ABS phantoms in the MC simulations. The CF(*r*) obtained from the ratio between both profiles is also depicted. All profiles were normalized with the calculated dose at a radial distance of 1 cm from the virtual source for water. The percentage dose for the radial distance of 1 cm was 93.20% in ABS, being lower than that in water. In addition, the acquired profiles for water were higher than those for ABS up to a radial distance of 2.1 cm and smaller for radial distances above 2.1 cm. The percentage difference between both profiles was 47% at a radial distance of 0.5 cm and gradually decreased until the difference between profiles can be negligible. At a radial distance above 1.5 cm, the difference was below 1%.

The CF(*r*) was 0.88 at a radial distance of 0.5 cm and gradually increased until a radial distance of 6 cm, reaching 1.08 [Figure 3 (bottom)]. At a radial distance above 6 cm, the value slowly decreased, reaching 1.04 at 8 cm. For both materials, the CF(*r*) was 1 at a radial distance of 2.1 cm. In this study, the values of CF(*r*) were only obtained within 8 cm because the percentage dose difference between both profiles at radial distances above 8 cm was negligible (< 1.5%).

### Validation of the Developed Dosimetric Verification System

**Figure 4:**
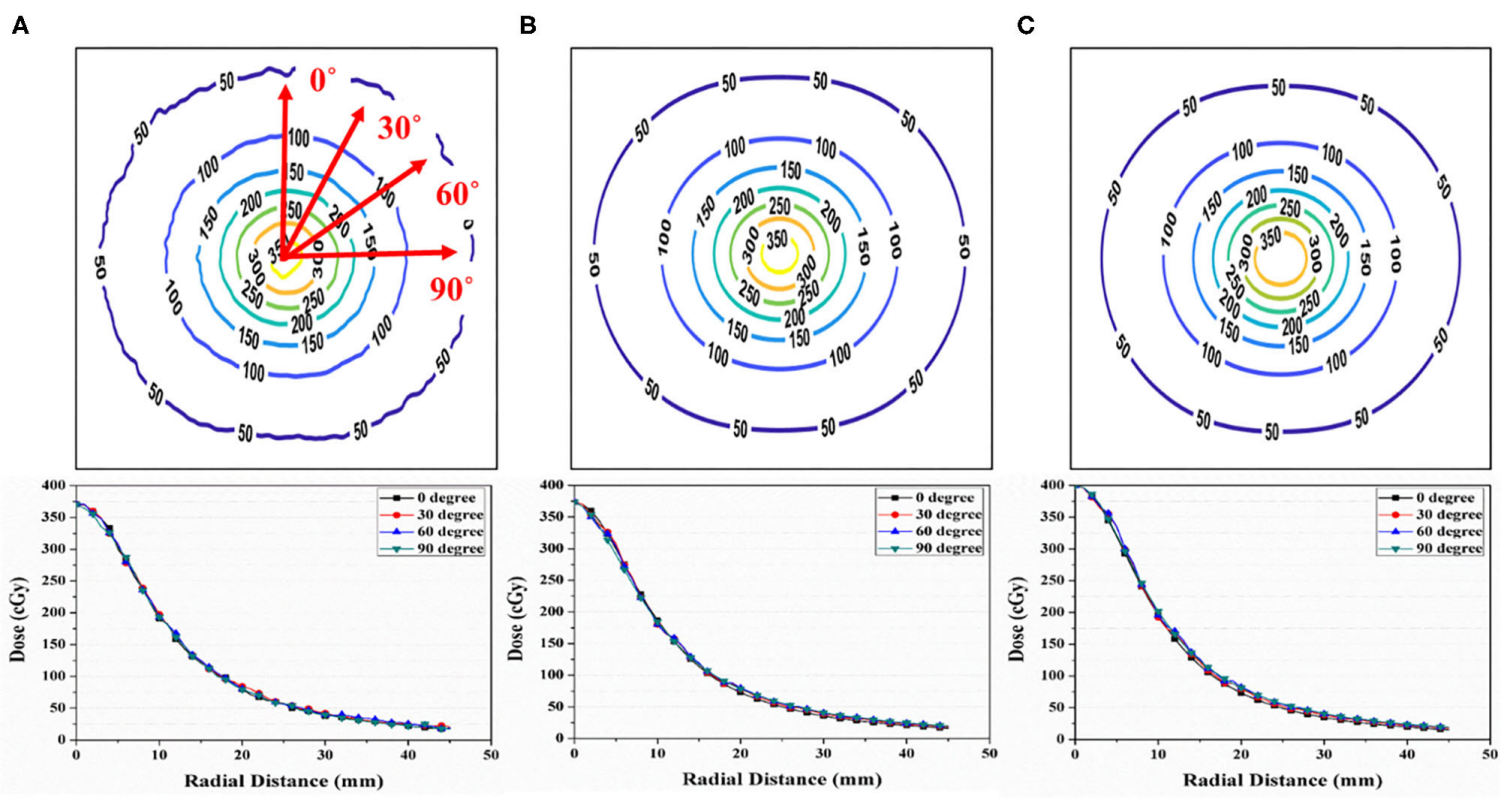
**(A)** Isodose map from EBT3 film measurement. **(B)** Independent dose calculation program (IDCP) and **(C)** treatment planning system (TPS) results at a single dwell position and 400 cGy. Each dose profile was obtained at angles of 0, 30, 60, and 90°.

Figure 4 shows the isodose map obtained from the film measurements, IDCP, and TPS at a single dwell position. The central point dose for each dose distribution was 3.70 Gy in film measurements, 3.73 Gy for the IDCP, and 4 Gy for the TPS. The IDCP and film measurement profiles were similar. Although the TPS profile was higher than the other profiles within 0.8 cm from the center of the isodose map, there was no considerable difference in each profile according to the angle.

**Figure 5:**
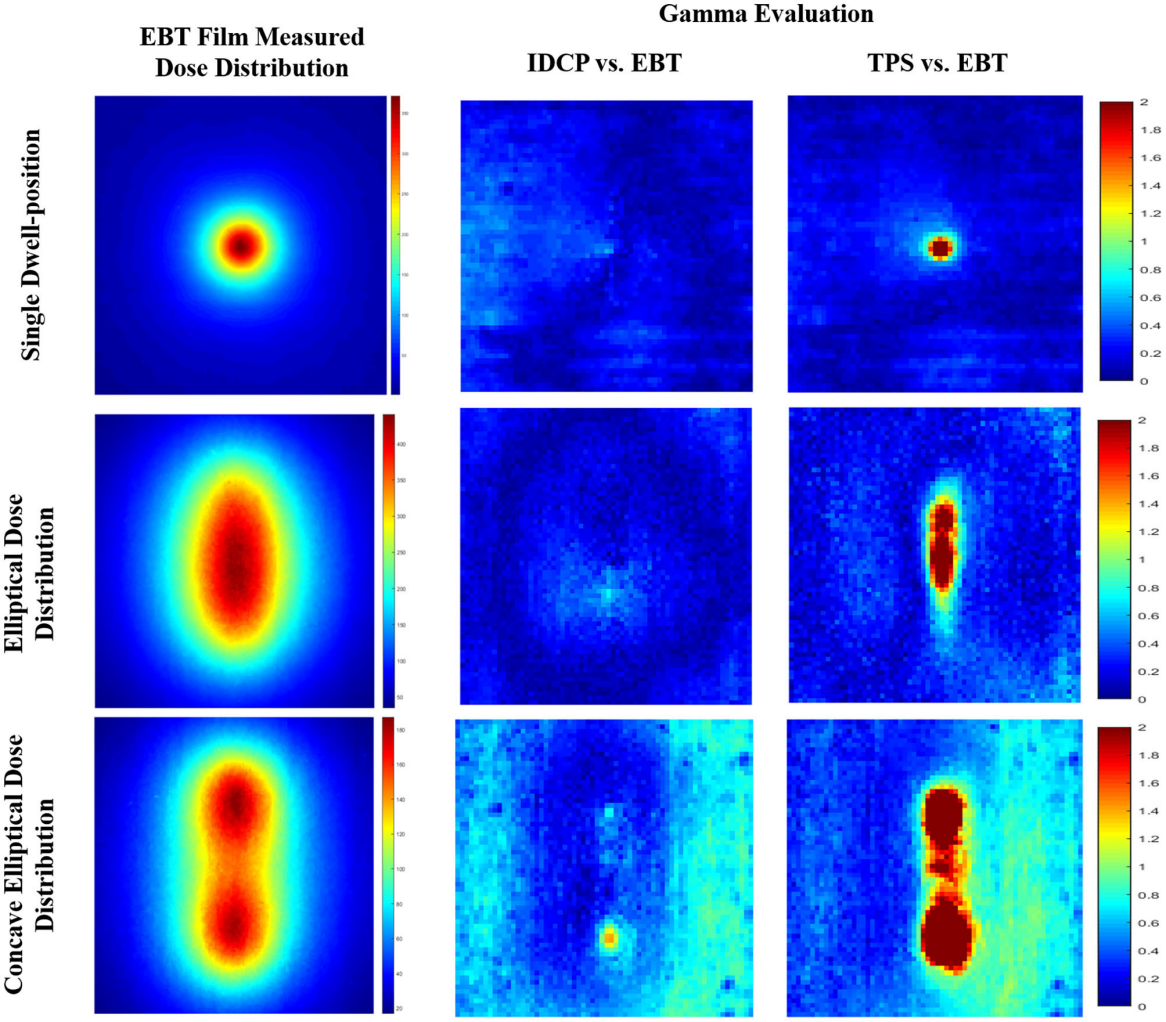
Gamma analysis between measured and calculated dose distributions using 3%/3 mm criterion for three simulated cases: single dwell position, elliptical dose distribution, and concave elliptical dose distribution.

Figure 5 shows the gamma analysis between the measured and calculated dose distributions for the three simulated cases. Based on the measured dose distribution at a single dwell position, the gamma passing rates using the 3%/3 mm criterion were 99.41 and 100% for the TPS and DVS, respectively. In the dose distribution near the source, there was a lower passing rate in the TPS than in the DVS. For the elliptical dose distribution, the passing rates analyzed with 3%/3 mm criterion were 96.80 and 100% for the TPS and DVS, respectively. The gamma failure of the TPS was higher than that of the DVS near the source. For the concave elliptical dose distribution, the gamma passing rates were 88.91 and 99.70% for the TPS and DVS, respectively.

**Figure 6:**
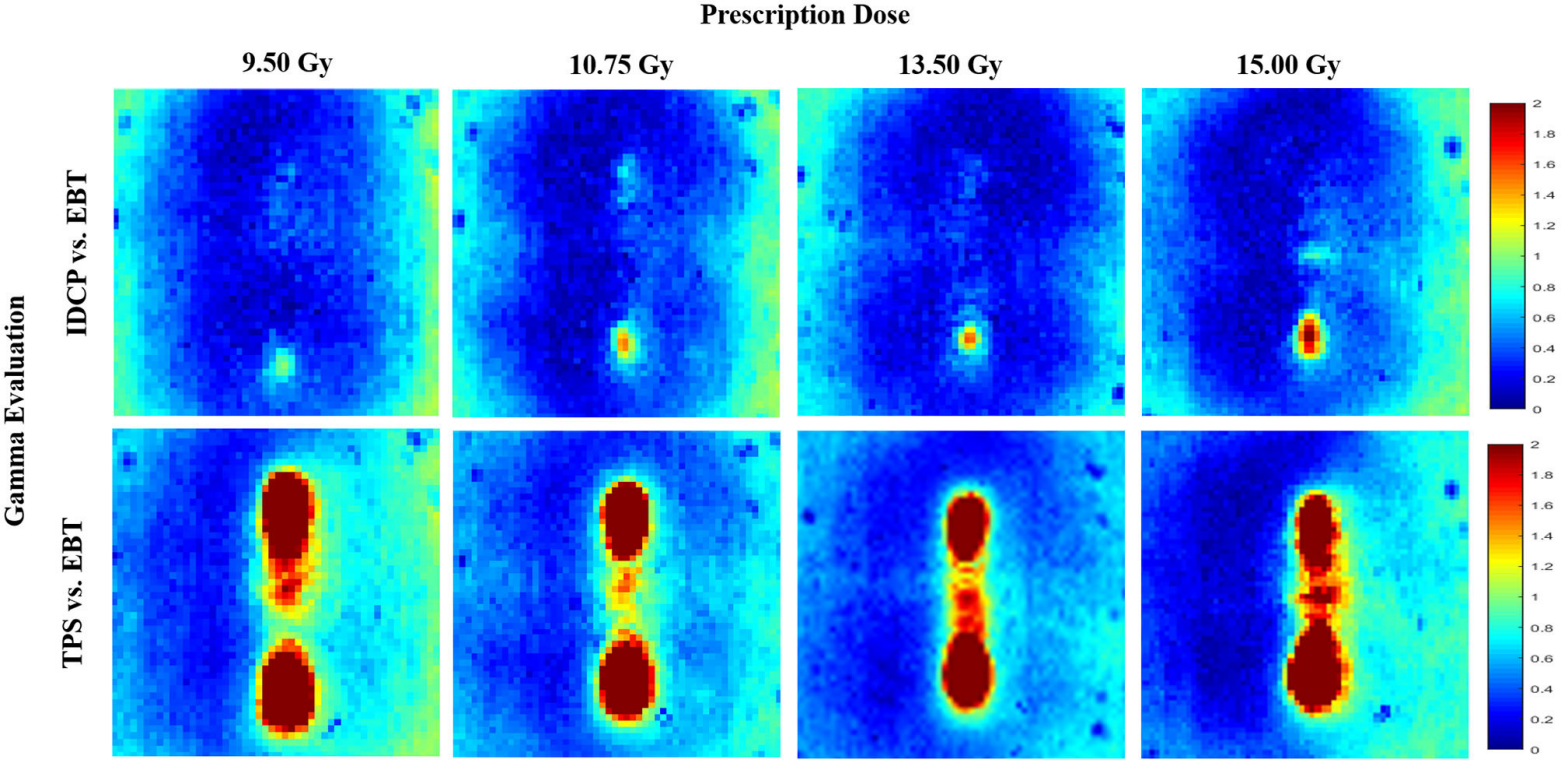
Gamma analysis between measured and calculated dose distributions using 3%/3 mm criterion for concave elliptical dose distribution at high doses.

Figure 6 shows the gamma analysis for the concave elliptical dose distributions at high doses. The gamma passing rates using the 3%/3 mm criterion in the dose distributions between the DVS results and measurements were 98.12, 98.32, 99.68, and 98.36% for 9.50, 10.75, 13.50, and 15 Gy, respectively. For these doses, the gamma passing rates in the dose distributions between the TPS results and measurements were 84.76, 89.71, 91.92, and 89.75%, respectively. Compared with the measured distributions, the dose distributions calculated by the DVS have higher gamma passing rates than those calculated by the TPS at all the high doses.

## Discussion

Accurate QA procedures for HDR-BT are important to increase the likelihood of desired treatment outcomes, minimize the risk of errors in clinical practice, and ensure the efficacy of clinical trials. In previous studies (11–14), these dosimetric verifications were able to perform point-dose measurements using the specific water phantom or Solid Water^TM^ phantom. However, these QA procedures had limitations in its practical application because there were inconvenient to use a specific water phantom and were insufficient to verify the treatment plan with only point-dose measurement. Therefore, the DVS, which can easily apply QA and verify dose distribution as well as point-dose, was developed in this study.

The CF(*r*) is the most important factor for the DVS to perform the dosimetric verification with the solid-phantom. The concept of the CF has already been reported in previous studies (13, 14, 20). However, since the CF was obtained only at a certain-depth and was limitedly used to verify the point-dose, it was not applied to convert the dose distribution in the solid phantom. In this study, we determined the modified CF as a function of the radial distance, the CF(*r*), and applied it to the IDCP to calculate the accurate dose distribution in the solid phantom. Through the DVS validation, it has been proven that the QA procedure using IDCP and ABS solid phantom can be applied to the dosimetric verification of HDR-BT if the CF(*r*) is appropriately considered.

To investigate the feasibility of the DVS for QA of HDR-BT, we compared the gamma evaluations of the dose distributions calculated by the DVS/TPS with those measured using film for the three cases under various dose ranges. All gamma passing rates were higher in the dose distributions between the DVS results and measurements than in those between the TPS results and measurements. Thus, the dose distribution calculated by the DVS is more consistent with that measured by the film. In addition, the results suggest that the DVS establishes an effective verification method for complex dose distributions using high dose ranges. Thus, we believe that the DVS can be used as a QA tool for pretreatment verification in HDR-BT.

We consider that the DVS can support the verification of the dosimetric parameters of the source and the QA of HDR-BT. If dosimetric parameters such as radial dose and anisotropic functions are not accurate, the dose distribution cannot be calculated correctly. This can be simply verified by comparison with the measured dose distribution at a single dwell position. In addition, the evaluation of the elliptical dose distribution can support various QA procedures for HDR-BT, such as source position verification, timer accuracy, and linearity testing.

For pretreatment verification of HDR-BT, the DVS was derived from patient-specific QA used in external beam radiotherapy delivery techniques, such as intensity modulated radiation therapy and volumetric modulated arc therapy. The HDR-BT treatment plan was established by determining the dwell position of the source with respect to the shape of the applicator and using multiple catheters depending on the number of channels. To perform pretreatment verification in the DVS, the complex dwell position of each channel was modified to a linear dwell position that fitted the catheter hole in the solid phantom. Thus, the verification of the treatment plan for HDR-BT can be performed using the DVS and the solid phantom. However, the treatment plan converter was not applied in this study because only a simulated plan instead of a clinical plan was used. In a future study, we will perform dosimetric verification in clinical cases using the DVS to apply the treatment plan converter.

To evaluate the DVS performance, film dosimetry was used in this study. Although films are generally energy dependent, some studies have demonstrated that the EBT3 film can be used in dose measurements for HDR-BT. Parmer et al. (11) reported the successful application of the EBT3 film to dose measurements in HDR-BT. In addition, Devic et al. (21) noted that the energy range response of the film does not change by more than 0.3%. Consistent with previous studies, the DVS relies on the EBT3 film to verify treatment plans generated at high doses as well as doses for actual clinical conditions.

## Conclusion

The proposed DVS is applicable for dosimetric verification, as demonstrated through simulated cases at various doses. From this study, we believe that the DVS can be used for QA of HDR-BT and to deliver more accurate and safer treatments. In addition, this study showed the possibility of performing patient-specific QA of HDR-BT using a solid phantom instead of a water phantom if CF(*r*) is correctly determined.

## Data Availability Statement

The original contributions presented in the study are included in the article/supplementary material, further inquiries can be directed to the corresponding author/s.

## Author Contributions

J-BC and TS supervised the project. S-WK, SK, and JP conceived and designed the experiments. CC, H-JP, K-YE, J-YK, CS, IK, and J-SK. contributed the experiments. S-WK, K-HK, SK, D-SS, and WC built the in-house software. S-WK, J-BC, and JP wrote the manuscript. All authors contributed to the article and approved the submitted version.

## Conflict of Interest

The authors declare that the research was conducted in the absence of any commercial or financial relationships that could be construed as a potential conflict of interest.
